# Changes in the Use of Fresh-Frozen Plasma Transfusions in Preterm Neonates: A Single Center Experience

**DOI:** 10.3390/jcm9113789

**Published:** 2020-11-23

**Authors:** Nina A. M. Houben, Lisanne E. Heeger, Simon J. Stanworth, Helen V. New, Johanna G. van der Bom, Suzanne Fustolo-Gunnink, Enrico Lopriore

**Affiliations:** 1 Division of Neonatology, Department of Pediatrics, Leiden University Medical Center, 2333ZA Leiden, The Netherlands; n.a.m.houben@lumc.nl (N.A.M.H.); l.e.heeger@lumc.nl (L.E.H.); 2 Radcliffe Department of Medicine, University of Oxford, and Oxford BRC Haematology Theme, Oxford OX3 9BQ, UK; simon.stanworth@nhsbt.nhs.uk; 3 Clinical Directorate, NHS Blood and Transplant, London NW9 5BG, UK; Helen.New@nhsbt.nhs.uk; 4 Department of Haematology, Imperial College London, London W12 0NN, UK; 5 Center for Clinical Transfusion Research (CCTR) LUMC/Sanquin, 1066CX Amsterdam, The Netherlands; j.g.van_der_bom@lumc.nl (J.G.v.d.B.); s.f.gunnink@amsterdamumc.nl (S.F.-G.); 6 Department of Clinical Epidemiology, Leiden University Medical Center, 2333ZA Leiden, The Netherlands

**Keywords:** fresh-frozen plasma transfusion, coagulation testing, preterm neonates, neonatal intensive care unit, single center experience

## Abstract

The aim of this study was to evaluate changes in the use of fresh-frozen plasma (FFP) transfusions and the use of clotting tests in preterm neonates in our center over the past two decades. In this retrospective cohort analysis, we included all consecutive neonates with a gestational age at birth between 24 + 0 and 31 + 6 weeks admitted to our neonatal intensive care unit (NICU) between 2004 and 2019. We divided all included neonates into three consecutive time epochs according to date of birth: January 2004 to April 2009, May 2009 to August 2014 and September 2014 to December 2019. The main outcomes were the use of FFP transfusion, coagulation testing and the indications for FFP transfusion. The percentage of preterm neonates receiving FFP transfusion decreased from 5.7% (47/824) to 3.7% (30/901) to 2.0% (17/852) from the first epoch to the last epoch (*p* < 0.001). Additionally, the rate of neonates undergoing coagulation testing decreased from 24.3% (200/824) to 14.5% (131/901) to 8% (68/852) over the epochs (*p* < 0.001). Most FFP transfusions were prescribed prophylactically based on prolongation of activated partial thromboplastin time (aPTT) or prothrombin time (PT) (56%). In conclusion, both the use of FFP transfusions and the use of coagulation tests decreased significantly over the years. The majority of the FFP transfusions were administrated prophylactically for abnormal coagulation tests.

## 1. Introduction

Preterm neonates are a highly vulnerable group of patients at risk of severe bleeding complications such as intraventricular hemorrhages (IVH). Various treatments are used to try to prevent the occurrence of hemorrhages, including the administration of platelets, vitamin K and fresh frozen plasma (FFP) [1,2]. Although FFP transfusions are often given ‘prophylactically’ in non-bleeding neonates, their use is not based on robust evidence [3,4]. To date, randomized trials have failed to show a beneficial effect of FFP transfusion on bleeding risk [5]. Despite the lack of evidence, international guidelines often recommend the use of FFP in neonates with clinically significant bleeding and those who have abnormal coagulation tests, or prior to invasive procedures [6,7,8,9]. Since these guidelines are mainly based on expert opinion and extrapolate findings from studies in adults, international consensus on the use of FFP is lacking, with wide variation in the use of FFP transfusion among Neonatal Intensive Care Units (NICUs). Reports on the proportion of neonates receiving FFP during admission to the NICU vary from 2 to 11% [3,10,11]. Not much is known on the variation of the use of FFP between neonatal centers or per indication, nor on the variation throughout the years. Neonatal coagulation ranges are different from those for older children and adults, and since it is unclear whether mild/moderate prolongation of PT and aPTT values predict clinical bleeding, interpreting neonatal coagulation test results can be difficult, which can contribute to variation and inappropriate use of FFP [3,12,13,14,15].

A better understanding of changes in and indications for the use of FFP in the past can contribute to more evidence-based practice in the future. Therefore, we aimed to evaluate changes in the use of FFP transfusions and clotting tests in preterm neonates in the past two decades at our center.

## 2. Materials and Methods

### 2.1. Study Design and Population

We conducted a retrospective cohort analysis in the NICU of the Leiden University Medical Center, one of 9 level III NICUs in The Netherlands. All neonates with a gestational age at birth between 24 + 0 and 31 + 6 weeks, admitted to our NICU in a 16-year period between 1 January 2004 and 31 December 2019, were included. We divided the neonates into three consecutive time periods (Epoch I, II and III) according to date of birth: January 2004 to April 2009, May 2009 to August 2014 and September 2014 to December 2019, respectively. The institutional review board of the Leiden University Medical Center approved the study and waived the need for informed consent.

### 2.2. Guidelines

The reference ranges used in our NICU for coagulation testing in preterm neonates with a gestational age between 30 and 36 weeks were based on a prospective cohort study by Andrew et al. (see Table A1 in Appendix A) [16]. Our guideline for FFP transfusion remained unchanged throughout the last two decades and stated that neonates without clinical bleeding tendency with abnormal coagulation values exceeding twice the mid-point of the reference values of activated partial thromboplastin time (aPTT) or prothrombin time (PT) should be treated with FFP transfusion (10 mL/kg over 30 to 60 min). Additionally, the guideline mentioned that these limits are not absolute (given the wide 95% confidence intervals), but also depend on the severity of the clinical condition. In the absence of reference ranges for premature neonates below 30 weeks, the values mentioned above also applied to these patients [17].

### 2.3. Outcome Measures

The main outcome measures were the percentage of neonates receiving FFP transfusion and the percentage of neonates in whom coagulation testing was performed per epoch. Additionally, we examined the primary indications for FFP transfusion as described in the patient records; abnormal coagulation was defined as at least one prolonged aPTT or PT test result exceeding the upper limit of the reference values by Andrew et al. during admission, and disseminated intravascular coagulation (DIC) was defined as the combination of a platelet count below 100 × 10^9^ platelets per liter, activated partial thromboplastin time (aPTT) >90 s, prothrombin time (PT) ratio >1.5, fibrinogen concentration below 150 mg/dl, no improvement after administration of vitamin K and absence of liver disease [16,18].

As an additional outcome, we assessed the occurrence of major hemorrhage per epoch. Major hemorrhage was defined as intraventricular hemorrhage ≥ grade 3, severe gastrointestinal hemorrhage (any amount of fresh visible rectal bleeding except for mild bleeding caused by necrotizing enterocolitis (NEC)) and pulmonary hemorrhage (defined as fresh bleeding through an endotracheal tube with increased ventilatory requirements) [19,20]. We also evaluated if undergoing coagulation testing was associated with higher odds of receiving FFP transfusion. Other additional outcomes included the number of neonates with abnormal coagulation, the percentage of neonates receiving FFP transfusion and the percentage of neonates undergoing coagulation testing per gestational age at birth in weeks. Baseline characteristics included gender, gestational age at birth, birth weight, small for gestational age (SGA) (birth weight < 10th centile), multiple birth and delivery mode [21]. We also collected the following neonatal outcome variables: NEC ≥ stage 2, proven sepsis defined as a clinically ill neonate with a positive bacterial blood culture, symptomatic patent ductus arteriosus (PDA) requiring medical treatment (indomethacin or ibuprofen) or surgical closure, respiratory distress syndrome (RDS) defined as respiratory failure requiring ventilator support and surfactant treatment, length of hospital stay in days and neonatal mortality during admission [22,23].

### 2.4. Statistical Analyses and Sample Size

All computations were performed using Statistical Package for the Social Sciences (SPSS) version 24.0. We used the Cochran–Armitage test for trend to examine changes in the use of plasma transfusions over three epochs, likewise for the use of coagulation testing. Normally distributed values are presented as means (standard deviation (SD)), and non-normal variables as medians (interquartile range (IQR)). We evaluated the possible association between undergoing coagulation testing and receiving FFP transfusion using logistic regression. A *p*-value of less than 0.05 is considered to be statistically significant.

## 3. Results

Between 1 January 2004 and 31 December 2019, 2577 preterm neonates with a gestational age <32 weeks were admitted to our center. The baseline characteristics of the neonates are shown in Table 1. Baseline characteristics and neonatal outcomes per epoch are presented in Figures S1 and S2, respectively.

**Figure 1:**
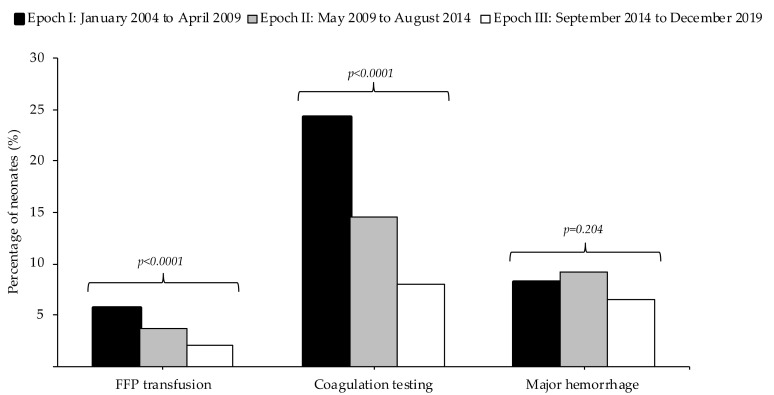
Plasma transfusions, coagulation testing and major hemorrhages in three epochs from 2004 to 2019.

A total of 94 patients (3.6%) received one or more FFP transfusions during admission. Among these 94 patients, the median number of FFP transfusions per neonate was 1 (IQR: 1–7). Coagulation testing was performed in 399 out of 2577 patients (15.5%), with a median number of coagulation tests per neonate of 1 (1–16). As presented in Figure 1, both the percentage of neonates receiving plasma transfusions and the percentage of neonates in which coagulation was determined decreased over the three epochs (*p* < 0.001). The percentage of neonates with major hemorrhage per epoch did not change significantly.

The indications for the use of FFP were summarized in Table 2. Most transfusions were administered prophylactically based on the prolongation of aPTT or PT, DIC (20.2%) or other causes of abnormal coagulation (56.3%).

Overall, 399 neonates underwent coagulation testing: aPTT and PT were prolonged in 367 (92.0%) and 256 (64.2%) of these neonates, respectively. Coagulation testing was performed in 200 of the 824 neonates in Epoch I (aPPT prolongation in 92.5%, PT prolongation in 64.5%); in 131 of the 901 neonates in Epoch II (aPPT prolongation in 90.8%, PT prolongation in 66.4%); in 68 of the 852 neonates in Epoch III (aPTT prolongation in 92.6%, PT prolongation in 30.5%).

A total of 94 neonates received FFP transfusion. Coagulation was determined in 87 out of the 94 (92.6%) FFP-transfused neonates: aPTT was prolonged in 85 of the 87 tested neonates (97.7%) and PT was prolonged in 80 of the 87 tested neonates (91.9%).

**Figure 2:**
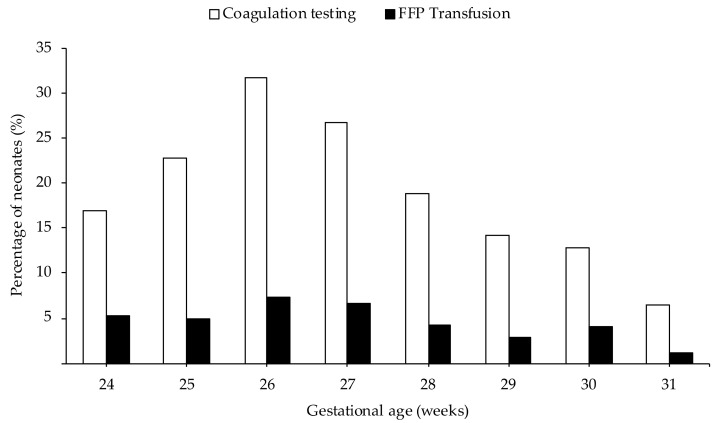
Percentage of neonates receiving FFP transfusion and percentage of neonates undergoing coagulation testing per gestational age at birth in weeks.

As presented in Figure 2, both FFP transfusions and coagulation tests were most frequently used in neonates born at 26 weeks of gestation and their use decreased with subsequent increasing gestational age at birth.

## 4. Discussion

In this retrospective study, we found that the percentage of neonates receiving FFP transfusion decreased significantly over time: from 5.7% of the neonates born between January 2004 and April 2009 to 3.7% between May 2009 and August 2014 to 2.0% between September 2014 and December 2019. To our knowledge, this is the first study assessing changes in the use of FFP over the years, as most studies on the use of blood products in neonates have focused on red blood cells (RBC) and platelet transfusion. It is therefore uncertain whether a similar decrease also occurred in other NICUs. In the past few decades, the use of RBC and platelets has decreased, after studies showed that restrictive transfusion guidelines were non-inferior to liberal guidelines [19,24,25]. The reduced use of blood products (RBC and platelets) may have contributed to a concomitant decrease in the use of FFP transfusion.

Overall, we found that 94 (3.6%) preterm neonates admitted to our NICU received at least one FFP transfusion. A study by Keir et al. reported much higher use of FFP in 11% of the neonates [26]. This could be explained by the fact that they excluded neonates with a gestational age of >30 weeks. As shown in Figure 2, we found that the use of FFP and coagulation tests was lower in neonates with a gestational age at birth of 30 to 32 weeks.

Importantly, we also saw a decrease in the percentage of neonates undergoing coagulation testing per epoch, decreasing from 24.3% to 8% over the years. We speculate that this is probably partly due to the increased awareness of iatrogenic anemia in preterm neonates resulting from cumulative blood loss due to frequent blood draws for laboratory tests [27,28]. Health professionals may, therefore, have had a more conscious attitude towards the reduction in coagulation testing. In addition, it has been recognized that neonatal coagulation test results can be ambiguous and difficult to interpret in the absence of evidence-based reference values. Some guidelines express the aim to reduce routine neonatal coagulation testing, which may have made neonatologists more inclined to skip coagulation tests [6].

Most of the FFP transfusions in our NICU were administered prophylactically for abnormal coagulation tests and no bleeding. Therefore, the reduction in coagulation testing may partly explain the reduction in FFP administration over the epochs. Several studies likewise reported that abnormal coagulation was the most commonly used indication for FFP in the NICU [4,10,11,29], while some studies reported otherwise [3,30].

Although most FFPs in our study were administrated for prolonged coagulation (56%), there are several concerns about the use of coagulation values in preterm neonates. Firstly, it is unclear whether FFP transfusions can effectively correct the coagulation abnormalities and/or reduce the risk of clinical bleeding. Studies by Altuntas et al. and Puetz et al. found that FFP corrected the prolonged coagulation values in 40–60% of the neonates [10,11]. In contrast, FFP failed to correct coagulation abnormalities in 70% of the neonates in Johnson et al. [31]. Secondly, there is no consensus on whether prolonged coagulation predicts bleeding in preterm neonates. As no randomized trials have been conducted to substantiate this, only results from observational studies are available, in which some studies suggest that prolonged PT and aPTT values do not predict clinical bleeding [3,12], while others indicate that abnormal coagulation values are associated with an increased risk of bleeding [13,14,15]. Thirdly, the reference values for aPPT and PT currently used in the NICU are based on limited and mostly outdated evidence. These values often have very wide 95% confidence intervals for the upper and lower limit of normal. The analyzers and reagents used in the NICU may vary from those used to derive the published reference values. In the absence of normal values tailored to the equipment used per NICU, it can be difficult to interpret coagulation results [12,16,32]. Additionally, the reference values are often not validated for extreme prematurity below 30 weeks [16].

It is also unclear whether FFP transfusions could be effective in treating preterm neonates with DIC, the second most common indication in our study. A small randomized trial by Gross et al. previously showed that treatment with FFP yielded no difference in the outcome of DIC [18]. However, a recent study by Go et al. showed that FFP in combination with recombinant human soluble thrombomodulin could be beneficial for the treatment of neonatal DIC [33].

Finally, we also showed that FFP transfusions and coagulation tests were most frequently used in extremely preterm neonates (with the highest rate in neonates with a gestational age of 26 weeks at birth), which can be explained by the severe comorbidity often associated with extreme prematurity. As shown in this study, the use of FFP and coagulation tests further decreased with increasing gestational age. However, we found that the use of FFP and coagulation tests was lower in neonates born at 24 or 25 weeks of gestation. Neonatologists may have been reluctant to draw large blood samples from these extremely premature patients out of concern for iatrogenic anemia. An alternative explanation could be that since the risk of neonatal mortality is higher in extreme preterm neonates born at 24 or 25 weeks of gestation, these neonates may not have survived long enough to receive FFP transfusions [34,35].

International guidelines for FFP transfusions for neonates vary considerably from country to country, emphasizing a lack of consensus on the optimal use of FFP in preterm neonates [6,7,36,37,38,39]. As previously shown by Iorio et al. and Pavenski et al., these guidelines were often based on limited scientific evidence [8,9]. In the absence of an international evidence-based guideline for the use of FFP in preterm neonates, further research is needed to establish meaningful reference values to distinguish the neonates with prolonged aPTT or PT in which FFP transfusion can help to prevent bleeding.

This study has limitations including the retrospective nature of the study, as a result of which we were unable to register the indication for transfusion in 4 out of 94 neonates. Moreover, we have included the indications for the FFP transfusion as described in the patient file. If they recorded the FFP transfusion for abnormal coagulation, we assumed that this was in line with the guideline limit of twice the mid-point of the reference values. However, we did not differentiate between transfusions for abnormal coagulation that were prescribed in accordance with the guideline.

Secondly, aPTT was prolonged in about 90% of neonates when using the Andrews reference ranges [16]. We did not consider the severity of the coagulation abnormality in this study, indicating that some abnormal coagulation results may only be mild abnormalities. Without knowing the extent of the deviation of aPTT here, it may be difficult to interpret these results in terms of FFP transfusion decision making.

As this concerns a single center experience, the potentially limited applicability of our findings to other NICUs needs to be considered. Lastly, the reference values of PT and aPTT applied in our department may vary from those used in other institutions.

In conclusion, we found that the use of FFP transfusions and the number of coagulation tests decreased significantly over the years. This is an important finding as abnormal coagulation was the most frequent indication for FFP transfusion. Further research is needed because the use of coagulation values in preterm neonates is not well-validated, and whether FFP transfusion can effectively correct coagulation abnormalities or decrease bleeding risk is unknown.

## Figures and Tables

**Table 1:** Overall Baseline Characteristics.

	All Neonates (*n* = 2577)
Male gender, *n* (%)	1372 (53)
Multiple birth, *n* (%)	1013 (39)
Caesarean section, *n* (%)	1253 (49)
Small for gestational age, *n* (%)	224 (9)
Birth weight (g), median (IQR)	1252 (980–1520)
Gestational age at birth (weeks), median (IQR)	29 (28–31)

**Table 2:** Indications for FFP Transfusion.

	FFP Transfusion (*n* = 94)
Abnormal coagulation, *n* (%)	53 (56)
Prolonged PT and aPTT	52 (55)
Prolonged aPTT	1 (1)
DIC, *n* (%)	19 (20)
Clinical bleeding, *n* (%)	16 (17)
Indication not recorded, *n* (%)	4 (4)
Surgery, *n* (%)	1 (1)
Suspected abnormal coagulation	1 (1)

FFP = Fresh Frozen Plasma, PT = Prothrombin Time, aPTT = activated Partial Thromboplastin Time, DIC = Disseminated Intravascular Coagulation.

## Supplementary Materials

The following are available online at https://www.mdpi.com/2077-0383/9/11/3789/s1, Figure S1: Baseline characteristics per epoch, Figure S2: Neonatal outcomes per epoch.

## Appendix A

**Table A1:** Reference Ranges for Healthy Preterm Neonates (GA 30–36 weeks) [16].

Test	Day 1	Day 5	Day 30	Day 90
PT (s) (95% interval)	13.0 (10.6–6.2)	12.5 (10.0–15.3)	11.8 (10.0–13.6)	12.3 (10.0–14.6)
aPTT (s) (95% interval)	53.6 (27.5–79.4)	50.5 (26.9–74.1)	44.7 (26.9–62.5)	39.5 (28.3–50.7)

PT = Prothrombin Time, aPTT= activated Partial Thromboplastin Time.
