# Supplementary material for: Changes in the Use of Fresh-Frozen Plasma Transfusions in Preterm Neonates: A Single Center Experience
Source: J Clin Med. 2020 Nov 23;9(11):3789. doi: 10.3390/jcm9113789 (PMC7700187; doi:10.3390/jcm9113789)
Supplement: Supplementary file 1 [file jcm-09-03789-s001.pdf]

Supplementary Materials:

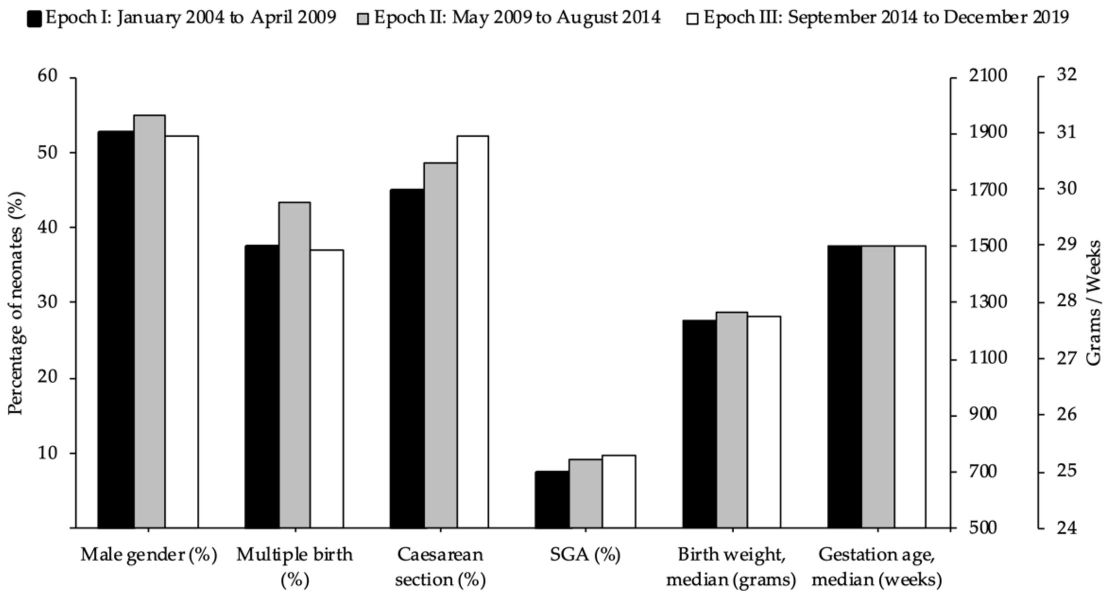

Figure S1. Baseline characteristics per epoch.

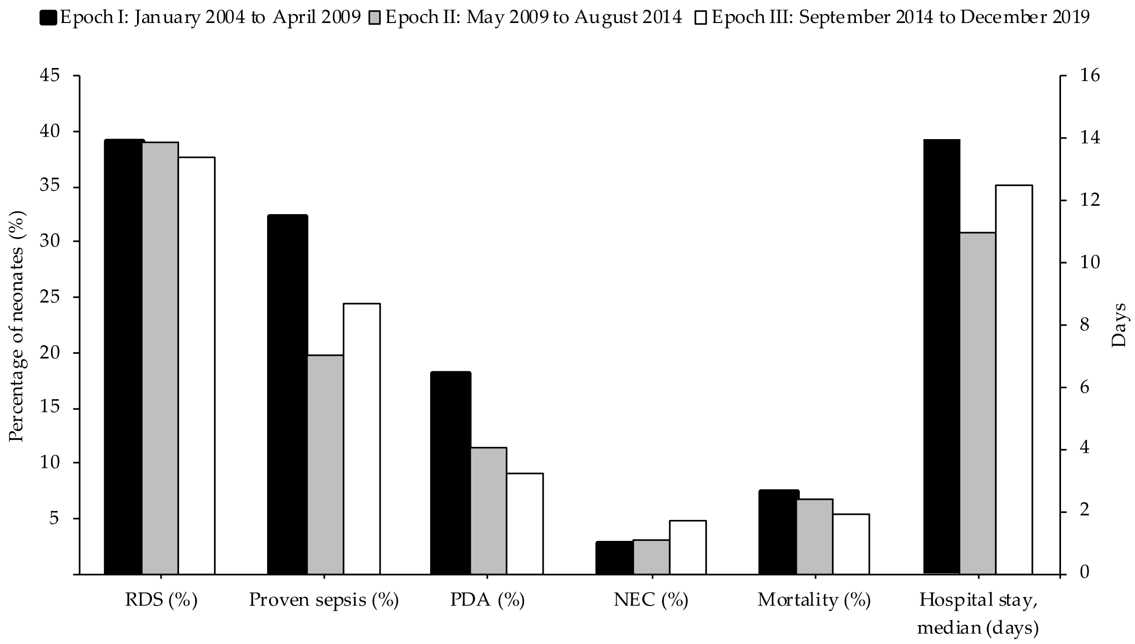

Figure S2. Neonatal outcomes per epoch.
